# Hospitalizations for People Aging with Schizophrenia

**DOI:** 10.1093/geroni/igaf122.3955

**Published:** 2025-12-31

**Authors:** Tracie Harrison, Cherisse Madlock-Brown, You Wang, Karen Dunn Lopez, Ryan Carnahan, Veronica Garcia Walker

**Affiliations:** University of Iowa College of Nursing, Iowa City, Iowa, United States; University of Iowa College of Nursing, Iowa City, Iowa, United States; University of Iowa College of Nursing, Iowa City, Iowa, United States; University of Iowa, Iowa City, Iowa, United States; The University of Iowa College of Public Health, Iowa City, Iowa, United States; The University of Texas at Austin, Austin, Texas, United States

## Abstract

Aging with schizophrenia or schizoaffective disorder is associated with high mortality. Quality of acute care in general hospital systems for older adults with long-term psychiatric conditions like schizophrenia and schizoaffective disorders is not well studied. After IRB approval, we extracted real-world data from 9,868 persons with a diagnosis of schizophrenia/schizoaffective disorder (62% female and 38% male) who received care in a midwestern, rural-designated hospital in the past 5 years. The majority were single and dismissed to self-care/home at an average age of 49 years. The length of stay for those over 65 (length of stay mean = 11.2 days) compared to under 65 (length of stay mean = 10.16) years of age was significantly (p = 0.008) higher. The length of hospitalization for men (11.97) was also higher than for women (9.35; p < 0.001). There were 17% with a 30-day readmission; the top readmission diagnoses included: shortness of breath, chest pain of unspecified type, and suicidal ideation, the same top three reason for initial admission. Chief complaints were non-specified in 42% of admissions. Over 93% of 30-, 60-, and 90-day readmissions were for reasons other than schizophrenia. Further explorations of quality care delivery, especially sex by age interactions for those with a diagnosis of schizophrenia, are needed.

